# 

**Published:** 1879-07

**Authors:** 


JULY, 1879.

PUBLISHED MONTHLY.

$2.50 PER ANNUM.

### THE AMERICAN JOURNAL OF DENTAL SCIENCE,

EDITED BY

F. J. S. GORGAS, M. D., D. D. S.,

AND

JAMES B. HODGKIN, D. D. S.

S.G.O.L. INDEXED

VOL. 13.—THIRD SERIES.—No. 3.

BALTIMORE:
SNOWDEN & COWMAN.

LONDON:

TRUBNER & CO., 60 PATERNOSTER ROW.

1879.

PAYABLE IN ADVANCE.

